# Skin colour and disease diagnosis: A cross‐sectional study of medical students in Kuwait

**DOI:** 10.1002/ski2.396

**Published:** 2024-05-13

**Authors:** Ghadeer Ahmad, Rudina Ghanem, Wafaa S. Mahfouz, Shoug AlHaddad, Wasmiyah AlHayyan, Amna AlMoosa, Hasan F. Shehab, Abdulmuhsen AlRasheid, Ahmad Garashi, Mohammad W. Kankouni, Ali H. Ziyab

**Affiliations:** ^1^ Department of Community Medicine and Behavioral Sciences College of Medicine Kuwait University Safat Kuwait

Dear Editor,

Dermatologic and systemic diseases show variation in their presentation on different skin colours; as a result, identification, diagnosis, and treatment of these diseases is proving to be challenging.^1^ Moreover, existing evidence shows inadequate representation of different dermatological manifestations in skin of colour (SoC) in the resources commonly used in medical training programs and by medical personnel.^2^ ^,^ ^3^ A previous study assessing the diversity of images used in preclinical anatomy textbooks found that light skin colour images comprised 74.5% of the total images, medium skin colour images comprised 21%, while dark skin colour images comprised 4.5%.^4^ Another study showed that only 14.9% of 1123 images in medical students’ resources were classified as SoC.^5^ This underrepresentation of SoC is also seen in dermatology journals.^6^ Due to this underrepresentation, it is expected that the ability and confidence of medical students to make a valid diagnosis across different skin colours may be compromised, as evidenced by prior studies conducted in the United States^7^ ^,^ ^8^ and Canada.^9^ Given the lack of such assessments in Middle Eastern settings where the populations are ethnically diverse, we sought to assess the ability of medical students at Kuwait University to visually identify dermatological manifestations in SoC and light skin as well as to determine students’ self‐rated confidence in their visual diagnostic abilities.

The target study sample included students in the preclinical program (2nd to 4th year) and clinical program (5th to 7th year) at Kuwait University, College of Medicine. The curriculum of the pre‐clinical years is organ‐system based that focuses on building the theoretical aspects of medical practice, including pathology, pharmacology, biochemistry, clinical medicine, and other related fields, with some bedside hospital teaching. During the clinical years, students undergo training in dermatology in year six of their studies. Hence, the exposure of students to conditions with dermatological manifestations is cumulative, with a dermatology‐focused training during year six of their medical training.

Students were invited to participate using a web‐based questionnaire that was distributed electronically to all eligible students from April 11th to 16th, 2022. The invitation text message asked participants to complete the questionnaire without referring to any resources. This study was approved by the Health Sciences Center Ethics Committee for Students Research at Kuwait University (No. 569/2022). The study questionnaire included 12 multiple choice questions (MCQs; ‘What is the diagnosis?’) assessing six conditions (Online Supporting Information Figure S1; chickenpox, erythema migrans [Lyme disease], psoriasis, systemic lupus erythematosus, basal cell carcinoma, and atopic dermatitis) on both light skin (Fitzpatrick skin phototype [SPT]: I‐III) and SoC (SPT: IV‐VI). Each of the diagnosis questions was paired with a corresponding 5‐point Likert scale assessing the students’ confidence in their diagnosis. This questionnaire design was adopted from a prior study.^9^

Statistical analyses were conducted using SAS 9.4 (SAS Institute). McNemar's test was used to compare the proportions of correct visual diagnosis (correct vs. incorrect) and students' confidence in their visual diagnosis (confident/very confident vs. not/slightly/somewhat confident) across skin colours (light skin vs. SoC). Moreover, the Cochran‐Armitage test for trend was used to test trends in proportions across years of study within each skin colour.

In total, 855 students were eligible to participate in the study, of whom 653 students (76.4%) were enrolled. Table 1 shows the frequency of correct visual diagnoses of skin conditions in both light skin and SoC in the total study sample, as well as according to the year of study. Overall, students showed a higher likelihood of accurately identifying dermatologic conditions in light skin compared to SoC. This trend was observed for four out of the six assessed diseases: chickenpox (74.6% vs. 47.9%, *p* < 0.001), erythema migrans (Lyme disease; 79.8% vs. 23.1%, *p* < 0.001), systemic lupus erythematosus (73.5% vs. 55.0%, *p* < 0.001), and basal cell carcinoma (51.6% vs. 24.0%, *p* < 0.001). There was no significant difference in the proportion of students correctly identifying psoriasis (71.2% in light skin vs. 67.4% in SoC, *p* = 0.099) and atopic dermatitis (51.3% in light skin vs. 47.2% in SoC, *p*_trend_ = 0.160) across skin colours. Moreover, increasing trends in the correct diagnosis over the years of study were observed (Table 1). For example, correct classification of basal cell carcinoma in light skin increased from 35.2% among 2nd year students to 83.1% among 7th year students (*P*_trend_ <0.001). An additional analysis showed that students in the clinical years of their training (5th to 7th year) compared to students in the pre‐clinical years of their training (2nd to 4th) were more likely to correctly identify skin conditions in both light skin and SoC (Online Supplementary Table S1).

**TABLE 1:** Frequency of correct visual diagnosis of skin diseases in light skin and SoC in the total study sample and according to year of study.

Disease	Correct diagnosis in the total study and according to year of study, % (*n*)								
	Skin colour	Total (*n* = 653)	2nd (*n* = 162)	3rd (*n* = 102)	4th (*n* = 121)	5th (*n* = 83)	6th (*n* = 108)	7th (*n* = 77)	*P*_trend_ ^b^
Chickenpox	Light skin	74.6 (487)	65.4 (106)	68.6 (70)	66.1 (80)	83.1 (69)	89.8 (97)	84.4 (65)	<0.001
	SoC	47.9 (313)	46.3 (75)	34.3 (35)	51.2 (62)	59.0 (49)	54.6 (59)	42.9 (33)	0.157
	*p* ^a^	<0.001	<0.001	<0.001	0.027	<0.001	<0.001	<0.001	
Erythema migrans (lyme disease)	Light skin	79.8 (521)	48.1 (78)	89.2 (91)	93.4 (113)	92.8 (77)	86.1 (93)	89.6 (69)	<0.001
	SoC	23.1 (151)	22.8 (37)	27.5 (28)	23.1 (28)	21.7 (18)	25.0 (27)	16.9 (13)	0.418
	*p* ^a^	<0.001	<0.001	<0.001	<0.001	<0.001	<0.001	<0.001	
Psoriasis	Light skin	71.2 (465)	39.5 (64)	65.7 (67)	84.3 (102)	83.1 (69)	88.9 (96)	87.0 (67)	<0.001
	SoC	67.4 (440)	45.7 (74)	67.7 (69)	72.7 (88)	74.7 (62)	74.1 (80)	87.0 (67)	<0.001
	*p* ^a^	0.099	0.258	0.763	0.023	0.127	0.006	1.00	
Systemic lupus erythematosus (butterfly rash)	Light skin	73.5 (480)	39.5 (64)	80.4 (82)	87.6 (106)	92.8 (77)	79.6 (86)	84.4 (65)	<0.001
	SoC	55.0 (359)	28.4 (46)	56.9 (58)	70.3 (85)	67.5 (56)	59.3 (64)	64.9 (50)	<0.001
	*p* ^a^	<0.001	0.022	<0.001	0.001	<0.001	0.001	0.011	
Basal cell carcinoma	Light skin	51.6 (337)	35.2 (57)	39.22 (40)	34.7 (42)	65.1 (54)	74.1 (80)	83.1 (64)	<0.001
	SoC	24.0 (157)	21.6 (35)	19.6 (20)	13.2 (16)	28.9 (24)	32.4 (35)	35.1 (27)	0.001
	*p* ^a^	<0.001	0.005	0.004	<0.001	<0.001	<0.001	<0.001	
Atopic dermatitis (eczema)	Light skin	51.3 (335)	36.4 (59)	45.1 (46)	44.6 (54)	49.4 (41)	78.7 (85)	64.9 (50)	<0.001
	SoC	47.2 (308)	48.8 (79)	53.9 (55)	50.4 (61)	47.0 (39)	42.6 (46)	36.4 (28)	0.032
	*p* ^a^	0.160	0.041	0.233	0.385	0.758	<0.001	0.002	

Abbreviation: SoC, skin of colour.

^a^ *p*‐value calculated using McNemar's test for paired binary data to compare proportion of correct diagnosis across skin colours.

^b^ *p*‐value calculated using Cochran‐Armitage test for trend to assess linear trends in correct diagnosis proportions across years of study.

Table 2 shows the frequency of students' self‐reported confidence (confident/very confident) in their diagnosis of skin conditions according to skin colour in the total study sample and across years of study. For all of the assessed skin conditions, students were more likely to be confident/very confident when visually diagnosing skin conditions in light skin than in SoC. For example, 54.4% of students were confident/very confident in diagnosing psoriasis in light skin compared to 30.9% being confident/very confident in diagnosing psoriasis in SoC (*p* < 0.001). Analysis assessing trends showed that students' confidence in their visual diagnosis increased for all of the assessed skin conditions across years of study (Table 2).

**TABLE 2:** Students' self‐reported confidence (confident/very confident) in the visual diagnosis of skin diseases in light skin and SoC in the total study sample and according to year of study.

Disease	Confident/very confident in the total study and according to year of study, % (*n*)								
	Skin colour	Total (*n* = 653)	2nd (*n* = 162)	3rd (*n* = 102)	4th (*n* = 121)	5th (*n* = 83)	6th (*n* = 108)	7th (*n* = 77)	*P*_trend_ ^b^
Chickenpo*x*	Light skin	57.2 (374)	35.8 (58)	49.0 (50)	47.9 (58)	74.7 (62)	79.6 (86)	77.9 (60)	<0.001
	SoC	25.0 (163)	14.2 (23)	18.6 (19)	14.0 (17)	21.7 (18)	49.0 (53)	42.9 (33)	<0.001
	*p* ^a^	<0.001	<0.001	<0.001	<0.001	<0.001	<0.001	<0.001	
Erythema migrans (lyme disease)	Light skin	58.0 (379)	20.4 (33)	64.7 (66)	70.3 (85)	75.9 (63)	71.3 (77)	71.4 (55)	<0.001
	SoC	13.2 (86)	6.2 (10)	7.8 (8)	13.2 (16)	18.1 (15)	21.3 (23)	18.2 (14)	<0.001
	*p* ^a^	<0.001	<0.001	<0.001	<0.001	<0.001	<0.001	<0.001	
Psoriasis	Light skin	54.4 (355)	22.8 (37)	35.3 (36)	60.3 (73)	71.1 (59)	82.4 (89)	79.2 (61)	<0.001
	SoC	30.9 (202)	13.0 (21)	16.7 (17)	33.1 (40)	45.8 (38)	50.0 (54)	41.6 (32)	<0.001
	*p* ^a^	<0.001	0.060	<0.001	<0.001	0.002	<0.001	<0.001	
Systemic lupus erythematosus (butterfly rash)	Light skin	60.6 (396)	23.5 (38)	69.6 (71)	65.3 (79)	85.5 (71)	77.8 (84)	68.8 (53)	<0.001
	SoC	32.6 (213)	7.4 (12)	27.5 (28)	39.7 (48)	48.2 (40)	41.7 (45)	52.0 (40)	<0.001
	*p* ^a^	<0.001	<0.001	<0.001	<0.001	<0.001	<0.001	0.027	
Basal cell carcinoma	Light skin	22.4 (146)	16.1 (26)	15.7 (16)	12.4 (15)	26.5 (22)	39.8 (43)	31.2 (24)	<0.001
	SoC	11.2 (73)	4.3 (7)	3.9 (4)	6.6 (8)	19.3 (16)	17.6 (19)	24.7 (19)	<0.001
	*p* ^a^	<0.001	0.003	0.024	0.328	0.115	0.001	0.347	
Atopic dermatitis (eczema)	Light skin	34.3 (224)	15.4 (25)	25.5 (26)	34.7 (42)	43.4 (36)	55.6 (60)	45.5 (35)	<0.001
	SoC	24.2 (158)	16.1 (26)	14.7 (15)	19.8 (24)	31.3 (26)	36.1 (39)	36.4 (28)	<0.001
	*p* ^a^	<0.001	0.574	<0.001	0.003	0.048	0.001	0.041	

Abbreviation: SoC, skin of colour.

^a^ *p*‐value calculated using McNemar's test for paired binary data to compare proportion of students' self‐reported confidence (confident/very confident vs. not/slightly/somewhat confident) across skin colours.

^b^ *p*‐value calculated using Cochran‐Armitage test for trend to assess linear trends in students' self‐reported confidence proportions across years of study.

Our findings showed skin colour‐related disparities in the students' visual diagnostic accuracy and confidence in assessing skin conditions, with overall higher diagnostic accuracy and confidence in light skin compared to SoC. Moreover, we observed increasing trends in the diagnostic accuracy and confidence across years of study. These observations are in agreement with prior studies that showed higher diagnostic accuracy of skin conditions in light skin compared to SoC.^7^ ^,^ ^9^ Such disparities could be attributed to the underrepresentation of skin manifestations in SoC compared to light skin in educational resources. A study from the University of Bristol, United Kingdom, showed that students who were exposed to the updated dermatology curriculum in 2020 (incorporating more teaching on SoC) compared to students who were not exposed to the updated dermatology curriculum were more confident and accurate in diagnosing conditions in SoC.^10^ Hence, to address the existing disparity and educational deficiency, there is a need for medical curricula to incorporate comprehensive textual and visual materials that represent racial differences in dermatology. Addressing these educational disparities is crucial for ensuring equitable healthcare outcomes.

The findings of this report should be interpreted in light of the following limitations. First, the picture that was used to assess systemic lupus erythematosus in SoC might hamper correct identification due to the fact that it barely shows the right side of the face and the typical malar rash may be difficult to identify from the picture (Online Supplementary Figure S1). Hence, this issue in the provided picture of systemic lupus erythematosus in SoC might have biased the results of this assessment. Moreover, the clinical manifestations of some of the assessed conditions are distinct (e.g., basal cell carcinoma vs. chickenpox), and therefore differential diagnosis is less likely, which might bias the results (over‐estimate correct identification).

## AUTHOR CONTRIBUTIONS

**Ghadeer Ahmad**: Conceptualization (equal); formal analysis (equal); methodology (equal); project administration (equal); writing – original draft (equal). **Rudina Ghanem**: Conceptualization (equal); formal analysis (equal); methodology (equal); project administration (equal); writing – original draft (equal). **Wafaa S. Mahfouz**: Conceptualization (equal); formal analysis (equal); methodology (equal); project administration (equal); writing – original draft (equal). **Shoug AlHaddad**: Conceptualization (equal); formal analysis (equal); methodology (equal); project administration (equal); writing – original draft (equal). **Wasmiyah AlHayyan**: Conceptualization (equal); formal analysis (equal); methodology (equal); project administration (equal); writing – original draft (equal). **Amna AlMoosa**: Conceptualization (equal); formal analysis (equal); methodology (equal); project administration (equal); writing – original draft (equal). **Hasan F. Shehab**: Conceptualization (equal); formal analysis (equal); methodology (equal); project administration (equal); writing – original draft (equal). **Abdulmuhsen AlRasheid**: Conceptualization (equal); formal analysis (equal); methodology (equal); project administration (equal); writing – original draft (equal). **Ahmad Garashi**: Conceptualization (equal); formal analysis (equal); methodology (equal); project administration (equal); writing – original draft (equal). **Mohammad W. Kankouni**: Conceptualization (equal); formal analysis (equal); methodology (equal); project administration (equal); writing – original draft (equal). **Ali H. Ziyab**: Conceptualization (equal); formal analysis (equal); methodology (equal); supervision (equal); writing – review & editing (equal)

## CONFLICT OF INTEREST STATEMENT

The authors declare no conflicts of interest.

## FUNDING INFORMATION

This research received no specific grant from any funding agency in the public, commercial or not‐for‐profit sectors.

## ETHICS STATEMENT

This study was approved by the Health Sciences Center Ethics Committee for Students Research at Kuwait University (No 569/2022). Participation in the study was voluntary and informed consent was obtained from all participants.

## Supporting information

Figure S1

Table S1

## Data Availability

The data underlying this article will be shared upon reasonable request to the corresponding author.
