# Supplementary material for: Skin colour and disease diagnosis: A cross‐sectional study of medical students in Kuwait
Source: Skin Health Dis. 2024 May 13;4(4):e396. doi: 10.1002/ski2.396 (PMC11297429; doi:10.1002/ski2.396)
Supplement: Supplementary file 1 — Figure S1 [file SKI2-4-e396-s001.pdf]

Chickenpox

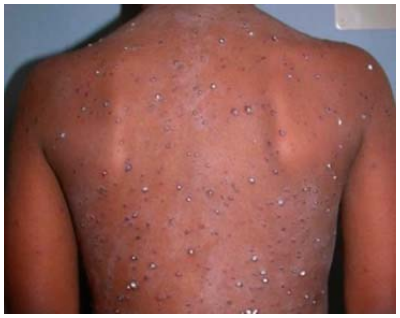

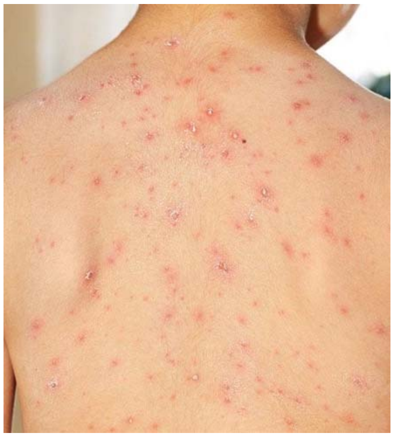

Lyme disease

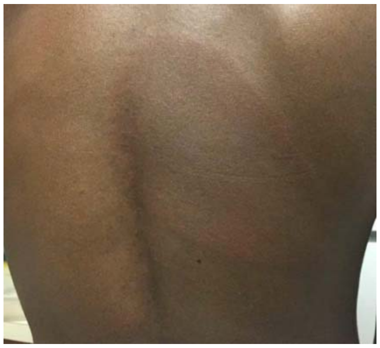

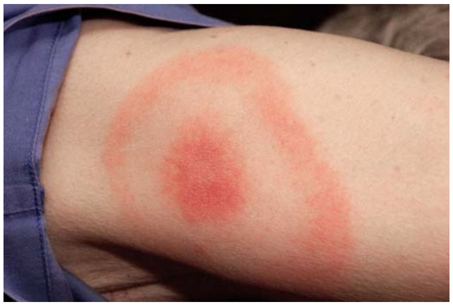

Psoriasis

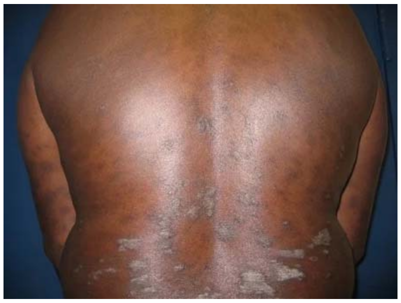

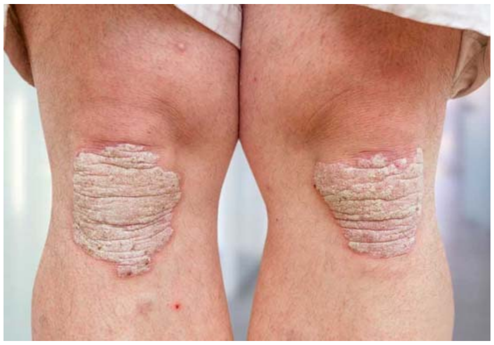

Systemic lupus erythematosus

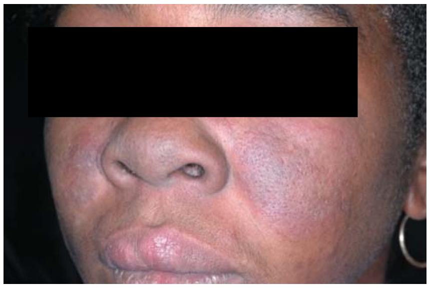

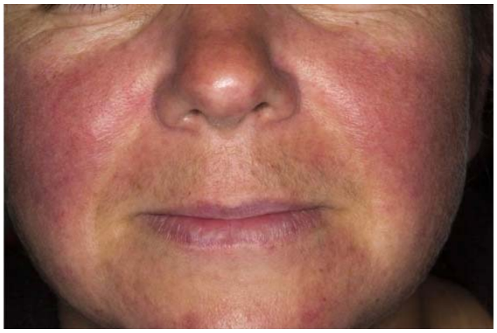

Basal cell carcinoma

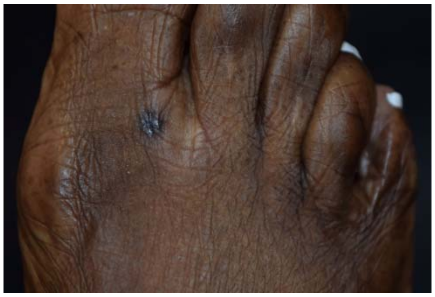

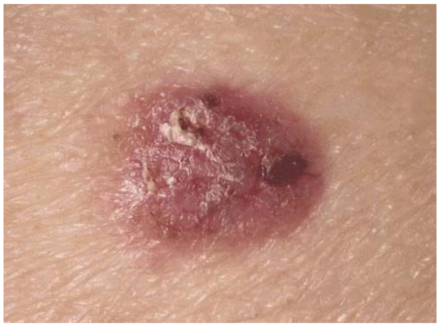

Atopic dermatitis (eczema)

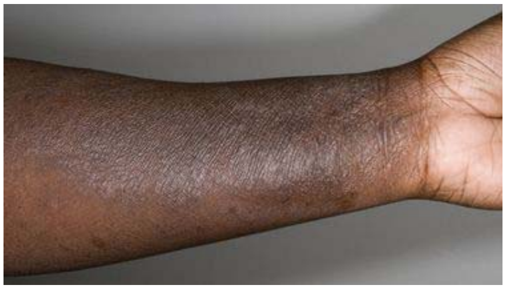

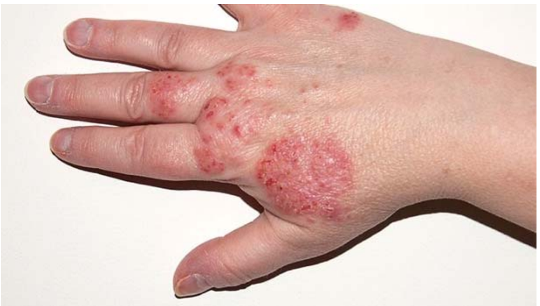

Figure S1. Images used for the assessment of skin manifestations in light skin and skin of color.
