# Supplementary material for: Skin colour and disease diagnosis: A cross‐sectional study of medical students in Kuwait
Source: Skin Health Dis. 2024 May 13;4(4):e396. doi: 10.1002/ski2.396 (PMC11297429; doi:10.1002/ski2.396)
Supplement: Supplementary file 2 — Table S1 [file SKI2-4-e396-s002.pdf]

Table S1. Frequency of correct visual diagnosis of skin diseases in light skin and skin of color (SoC) among preclinical years students (2nd to 4th year) and clinical years students (5th to 7th year)

		Correct diagnosis in preclinical and clinical years of study, % (n)		
**Disease**	**Skin color**	**Preclinical years (2nd to 4th year) (n = 385)**	**Clinical years (5th to 7th year) (n = 268)**	***P*†**
**Chickenpox**	Light skin	66.5 (256)	86.2 (231)	<0.001
	SoC	44.7 (172)	52.6 (141)	0.046
	P*	<0.001	<0.001	
**Erythema migrans (Lyme disease)**	Light skin	73.3 (282)	89.2 (239)	<0.001
	SoC	24.2 (93)	21.6 (58)	0.454
	P*	<0.001	<0.001	
**Psoriasis**	Light skin	60.5 (233)	86.6 (232)	<0.001
	SoC	60.0 (231)	78.0 (209)	<0.001
	P*	0.874	0.006	
**Lupus (Butterfly rash)**	Light skin	65.5 (252)	85.1 (228)	<0.001
	SoC	49.1 (189)	63.4 (170)	<0.001
	P*	<0.001	<0.001	
**Basal cell carcinoma**	Light skin	36.1 (139)	73.9 (198)	<0.001
	SoC	18.4 (71)	32.1 (86)	<0.001
	P*	<0.001	<0.001	
**Atopic dermatitis (eczema)**	Light skin	41.3 (159)	65.7 (176)	<0.001
	SoC	50.7 (195)	42.2 (113)	0.033
	P*	0.015	<0.001	

SoC: skin of color.

* *P*-value calculated using McNemar's test for paired binary data to compare proportion of correct diagnosis across skin colors.

† *P*-value calculated chi-squared test to assess differences in the frequency of correct diagnosis between students in the preclinical and clinical years of study of study.
